# Flame Retardancy of Vinyl Acetate–Ethylene Emulsion Adhesives Modified with Intumescent Flame Retardants and Fly Ash

**DOI:** 10.3390/polym18151884

**Published:** 2026-07-31

**Authors:** Qianqian Wang, Jing Luo, Yan Sun, Wei Liu, Xucai Wang, Peng Jiang

**Affiliations:** 1 National Key Laboratory of Strength and Structural Integrity, School of Aeronautic Science and Engineering, Beihang University, Beijing 100191, China; wqq2024@126.com; 2 Shandong Institute of Nonmetallic Materials, Jinan 250031, China; wangxucai01@163.com; 3 China Nuclear Power Engineering Co., Ltd., Beijing 100840, China; luojing@cnpe.cc; 4 Sichuan Fire Research Institute of Ministry of Emergency Management, Chengdu 610036, China; weiliu20141202@126.com; 5 Research Institute of Wood Industry, Chinese Academy of Forestry, Beijing 100091, China

**Keywords:** vinyl acetate–ethylene emulsion, fly ash, intumescent flame retardant, flame-retardant adhesive, char formation, solid waste utilization

## Abstract

Vinyl acetate–ethylene copolymer emulsion (VAE) adhesives are widely used in formaldehyde-free wood-based panels and coating systems, but their inherent flammability limits broader application in fire-safety-demanding fields. In this work, fly ash (FA), an industrial solid waste rich in Si- and Al-containing inorganic components, was introduced into a conventional ammonium polyphosphate/melamine/pentaerythritol (APP/MEL/PER) intumescent flame-retardant (IFR) system to prepare flame-retardant VAE emulsion adhesives. The results showed that the incorporation of IFR effectively improved the flame retardancy of VAE, while FA further enhanced the condensed-phase protective effect. The limiting oxygen index (LOI) of VAE/IFR/FA increased to 28.5% from 18.3% for neat VAE, and the sample achieved a UL-94 V-0 rating. Cone calorimetry results showed that the peak heat release rate and total heat release were significantly reduced after flame-retardant modification, accompanied by an increase in residual char. The VAE/IFR/FA sample exhibited the highest char residue and the lowest total smoke production, indicating the positive role of FA in promoting char formation and smoke suppression. The addition of KH403 further regulated the combustion behavior, mainly improving early-stage heat release suppression and fire performance index rather than increasing the final char yield. The KH403-containing formulation also showed improved tensile properties and plywood bonding strength, indicating its potential as a flame-retardant adhesive for wood-based panels. The KH403-containing formulation also showed improved tensile properties and plywood bonding strength, indicating its potential as a flame-retardant adhesive for wood-based panels. SEM-EDS, XPS, Raman, and TG-FTIR analyses confirmed that FA and KH403 contributed to the formation of a phosphorus-rich organic–inorganic hybrid char layer containing Si/Al inorganic structures. This reinforced char layer effectively inhibited heat transfer, oxygen diffusion, and the release of combustible volatiles. This study provides a feasible strategy for developing low-cost flame-retardant VAE adhesives while promoting the high-value utilization of fly ash.

## 1. Introduction

Poly (vinyl acetate) (PVAc) is inherently hard and brittle, limiting its application under ambient conditions [1]. Copolymerization with ethylene effectively improves the flexibility and durability of PVAc-based materials, leading to the development of ethylene-vinyl acetate copolymers (EVA) [2]. Due to their excellent flexibility, low-temperature resistance, corrosion resistance, and electrical insulation properties, EVA materials have been widely used in adhesives [3], coatings, packaging [4], construction, and energy-related fields [5,6,7,8]. The properties of EVA strongly depend on the vinyl acetate (VA) content. With increasing VA content, EVA gradually transforms from a thermoplastic resin into an elastomer, and eventually forms emulsion-type systems known as vinyl acetateethylene emulsions (VAE) [9].

VAE emulsions, typically prepared by emulsion polymerization of vinyl acetate and ethylene, exhibit excellent adhesion, water resistance, weather resistance, and film-forming properties. Therefore, they are widely used in construction adhesives, waterproof coatings, repair materials, and formaldehyde-free plywood manufacturing (since the adhesive layer can contribute to the fire safety of wood-based panels, flame-retardant VAE adhesives should be evaluated not only by their combustion behavior, but also by their bonding and mechanical performance) [10,11,12,13,14]. However, VAE is highly flammable because it mainly consists of carbon, hydrogen, and oxygen elements, exhibiting a low limiting oxygen index (LOI, ~19%) and poor UL-94 vertical burning performance [15,16,17]. During combustion, VAE undergoes rapid flame spread accompanied by intense heat release and smoke generation, which severely limits its application in building decoration, adhesive systems, and functional coatings [18]. Consequently, the development of efficient and environmentally friendly flame-retardant systems for VAE materials remains highly desirable.

Extensive studies have been conducted on the flame-retardant modification of EVA and VAE materials. Previous reports demonstrated that the incorporation of flame-retardant additives can effectively improve the fire safety of VAE systems. For example, VAE-based flame-retardant coatings significantly reduced the peak heat release rate (PHRR) and total heat release (THR) of combustible substrates while suppressing smoke generation. Since both VAE and EVA belong to ethylene-vinyl acetate copolymer systems and share similar acetate side-chain structures, they exhibit comparable thermal degradation behaviors, including side-chain decomposition, main-chain scission, and char formation [19]. Therefore, flame-retardant strategies developed for EVA can provide valuable guidance for VAE systems.

Among the reported flame-retardant approaches for EVA, inorganic flame retardants, phosphorus-containing flame retardants, and intumescent flame-retardant (IFR) systems have shown promising results. Xu et al. [20] reported that the synergistic combination of aluminum hydroxide (ATH) and melamine cyanurate (MCA) enabled EVA composites to achieve an LOI of 27.5% and a UL-94 V-0 rating. Phosphorus-containing flame retardants have attracted particular attention because of their high efficiency and environmental friendliness [21,22]. During combustion, phosphorus-containing compounds promote dehydration and carbonization of the polymer matrix, leading to the formation of dense char layers that effectively inhibit heat and mass transfer [23,24]. Yang et al. [25] further demonstrated that a hybrid system consisting of microencapsulated expandable graphite (MEG) and microencapsulated red phosphorus (MRP) significantly improved the flame retardancy of EVA, reducing the PHRR and THR by 91% and 74.3%, respectively. Similar effects have also been reported in VAE systems, indicating the considerable potential of phosphorus-based flame retardants for improving the fire safety of VAE emulsions.

Halogen-free intumescent flame retardants (IFRs) have received increasing attention due to their low toxicity, low smoke generation, and high flame-retardant efficiency [26,27,28]. Typical IFR systems consist of an acid source, a carbon source, and a blowing agent, which cooperate during combustion to form an expanded char layer that protects the underlying substrate from heat and oxygen transfer [29,30]. However, conventional IFR systems still suffer from limited flame-retardant efficiency and insufficient smoke suppression. Therefore, synergistic additives and functional fillers have been increasingly introduced to enhance their overall performance while reducing the loading level and minimizing adverse effects on material properties.

Fly ash (FA), a typical industrial solid waste generated from coal-fired power plants, mainly contains SiO_2_ and Al_2_O_3_ and possesses low density, high mechanical strength, and excellent thermal insulation properties [31]. In recent years, FA has attracted growing interest in fire-resistant and thermal insulation materials. Its inorganic components can improve thermal stability and char strength at elevated temperatures, while its utilization in flame-retardant systems is also beneficial for solid waste recycling. Previous studies have shown that FA can effectively improve the fire resistance of intumescent coatings and insulation materials [32,33]. Moreover, FA can form stable composite structures with polymer matrices and enhance the mechanical stability of the resulting materials. Nevertheless, the dispersion and interfacial compatibility of FA in polymer systems remain key factors influencing its flame-retardant performance [34,35]. Although APP/MEL/PER-based intumescent flame-retardant systems have been widely used in polymer materials, their efficiency in VAE emulsion adhesives still needs improvement, especially in terms of char stability and smoke suppression. Fly ash (FA), a Si/Al-rich industrial solid waste, has the potential to reinforce the condensed-phase char layer, but its poor compatibility with polymer matrices limits its effect. In this work, FA was introduced as a low-cost additive into the VAE/IFR system, and KH403 was used to improve the interfacial interaction among FA, IFR, and VAE. This design aims to enhance the flame retardancy of VAE adhesives while providing a feasible route for the high-value utilization of fly ash.

Based on these considerations, in this study, fly ash (FA) was combined with a conventional intumescent flame-retardant (IFR) system to prepare flame-retardant VAE emulsion adhesives. The combustion behavior of the prepared samples was systematically evaluated using limiting oxygen index (LOI) and cone calorimetry tests. In addition, thermogravimetric analysis, char residue characterization, and microstructural analyses were conducted to investigate the flame-retardant mechanism between FA and IFR. This work provides a feasible strategy for the high-value utilization of fly ash in flame-retardant VAE systems and offers theoretical support for the development of low-cost and environmentally friendly flame-retardant adhesives.

## 2. Materials and Methods

### 2.1. Materials

Vinyl acetate–ethylene copolymer emulsion (VAE, solid content: 55%) was supplied by GELIN HANYE Co., Ltd., Suzhou, China. Ammonium polyphosphate (APP, AR), melamine (MEL, AR), and pentaerythritol (PER, AR) were provided by Shandong Changsheng Flame Retardant New Material Co., Ltd., Dezhou, China. The intumescent flame retardant (IFR) was prepared by mixing APP, MEL, and PER at a mass ratio of 3:1:1. Silane coupling agent KH403, identified as 3-glycidoxypropyltrimethoxysilane (GPTMS), was purchased from Sinopharm Chemical Reagent Co., Ltd., Shanghai, China. Fly ash (FA) was supplied by Henan Jinhan Building Materials Co., Ltd., Zhengzhou, China; its morphology, apparent particle-size characteristics, and elemental composition determined by SEM–EDX are provided in the Supporting Information (Figure S2).

### 2.2. Preparation of Neat and Flame-Retardant VAE Samples

**Figure 1:**
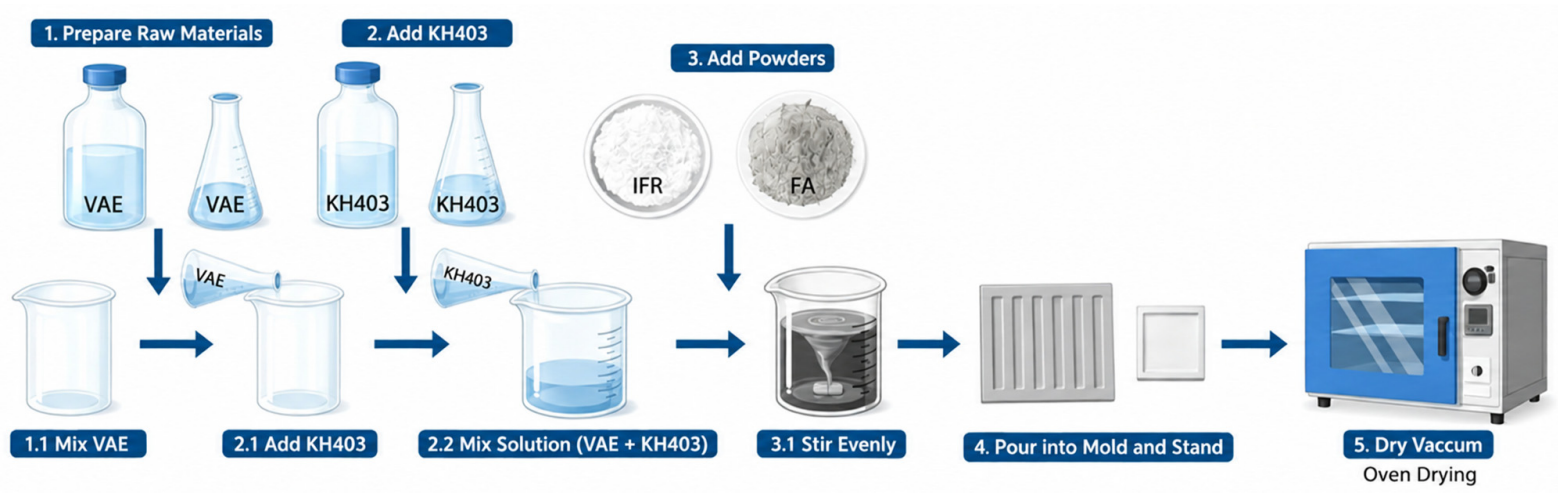
Schematic diagram of the preparation process for neat VAE (S1) and flame-retardant VAE samples (S2–S3).

The formulations of neat VAE and flame-retardant VAE samples are listed in Table 1. The intumescent flame retardant (IFR) was composed of APP, MEL, and PER at a mass ratio of 3:1:1. For the preparation of neat VAE, 100 g of VAE emulsion was directly used without adding any flame retardant or auxiliary agent. For the VAE/IFR sample, 75 g of VAE emulsion and 25 g of IFR were mixed using a high-speed disperser (LC-FS-400S, LICHEN company, Shaoxing, China) at 800 rpm for 10 min until a uniform dispersion was obtained. For the VAE/IFR/FA sample, 75 g of VAE emulsion, 20 g of IFR, and 5 g of fly ash were blended under the same conditions. For the VAE/IFR/FA/KH403 sample, 75 g of VAE emulsion, 20 g of IFR, 3 g of fly ash, and 2 g of KH403 were mixed to obtain a homogeneous adhesive system. After mixing, each formulation was poured into a mold and leveled with a scraper. The samples were first dried at 60 °C for 6 h to remove most of the water, and then further dried at 100 °C for another 6 h to obtain cured VAE-based samples (Figure 1). The resulting samples were denoted as S0 (Neat VAE), S1 (VAE/IFR), S2 (VAE/IFR/FA), and S3 (VAE/IFR/FA/KH403), respectively, according to the formulations shown in Table 1.

### 2.3. Characterization

The limiting oxygen index (LOI) was measured using an oxygen index tester (JF-3, Jiangning Analysis Instrument Co., Ltd., Nanjing, China) according to ASTM D2863-17. The specimen dimensions were 120 mm × 10 mm × 3 mm.

The combustion behavior of the samples was evaluated using a cone calorimeter (Fire Testing Technology Ltd., East Grinstead, West Sussex, UK) in accordance with ASTM E1354-17. Each specimen, with dimensions of 100 mm × 100 mm × 3 mm, was tested in a horizontal orientation under an external heat flux of 50 kW/m^2^. Before testing, the sides and bottom of each specimen were wrapped with aluminum foil, leaving only the upper surface exposed to the radiant heater. The wrapped specimen was placed on a ceramic backing board, with a distance of 35 mm from the cone heater.

The morphology of the char residues was observed using a scanning electron microscope (SEM, ZEISS Gemini SEM 300, Carl Zeiss Microscopy GmbH, Thuringia, Germany) equipped with an energy-dispersive X-ray spectroscopy (EDX) detector. Before observation, the char residues were sputter-coated with gold using an SCD005 sputter coater (BAL-TEC, Liechtenstein/Switzerland) at 40 mA for 180 s.

Raman spectroscopy was performed using a LabRAM HR Evolution Raman spectrometer (HORIBA, Paris, France) to evaluate the graphitization degree of the char residues. A 532 nm laser was used as the excitation source, and spectra were collected over the range of 50–4000 cm^−1^.

X-ray photoelectron spectroscopy (XPS) was conducted using a Thermo ESCALAB 250Xi system (Thermo Fisher Scientific, Waltham, MA, USA) equipped with an Al Kα X-ray source (hν = 1486.6 eV). The analysis chamber vacuum was maintained at 4 × 10^−9^ mbar. The working voltage and filament current were 14.6 kV and 13.5 mA, respectively. Spectra were acquired with 20 signal accumulation cycles, a pass energy of 20 eV, and a step size of 0.1 eV. The binding energies were calibrated using the C 1s peak at 284.8 eV.

The mechanical properties of the samples were measured using a CMT4104 universal testing machine (SANS, Shenzhen, China) according to ASTM D638. The tensile tests were carried out at a crosshead speed of 5 mm/min. The plywood bonding strength was determined according to GB/T 9846-2015 using specimens with dimensions of 100 mm × 25 mm × 15 mm. Five specimens were tested for each formulation, and the results are reported as mean ± standard deviation.

The UL-94 vertical burning test was performed using a CZF-3 vertical burning tester according to GB 4609-84. The test was used to evaluate the self-extinguishing behavior of the samples after ignition, as well as whether flaming drips could ignite the cotton indicator placed below the specimen. The specimen dimensions were 125 mm × 13 mm × 3 mm, and five specimens were tested for each formulation. According to the after-flame time, afterglow time, and dripping behavior, the samples were classified as V-0, V-1, V-2, or no rating.

## 3. Results

### 3.1. LOI Analysis

The LOI values and UL-94 ratings of neat VAE and flame-retardant VAE samples are shown in Table 2. Neat VAE (S0) exhibited the lowest LOI value of 18.3% and failed to obtain any rating in the UL-94 test, indicating its high flammability. After the incorporation of 25 wt% IFR, the LOI value of S1 (VAE/IFR) increased to 24.8%, and the sample achieved a V-1 rating. When fly ash (FA) was further introduced as additive, the LOI value of S2 (VAE/IFR/FA) increased markedly to 25.8%, corresponding to a 55.74% increase compared with S0. In addition, S2 passed the UL-94 V-0 test, suggesting that FA effectively enhanced the flame-retardant efficiency of the IFR system. For S3 (VAE/IFR/FA/KH403), in which FA and KH403 were added together, the LOI value reached 26.3%, representing a 43.72% increase over neat VAE, and a V-0 rating was also achieved. Among all samples, S2 showed the highest LOI value, indicating that the combination of FA and IFR was more effective in improving the flame retardancy of VAE. The improved fire performance may be related to the interaction between the oxygen-containing VAE matrix and the phosphorus-containing IFR system during combustion, which will be further discussed based on the char residue analysis and combustion mechanism.

### 3.2. Cone Calorimeter Analysis

**Figure 2:**
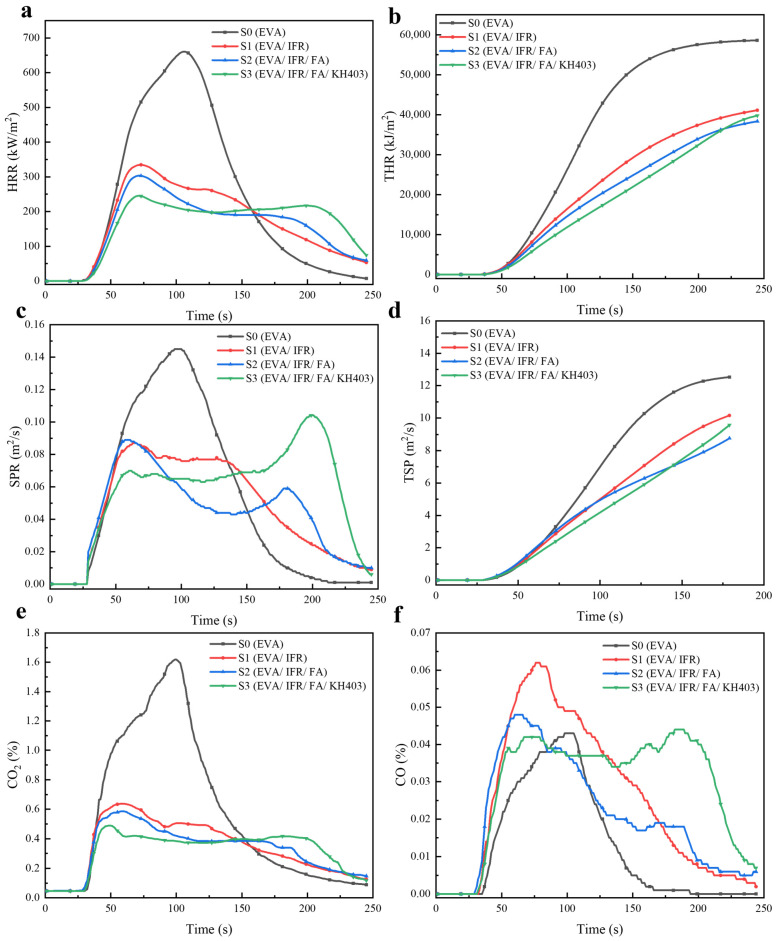
Heat release rates (**a**), total heat residual weight (**b**), smoke production rate (**c**) and total smoke production (**d**), CO_2_ concentration (**e**), CO concentration (**f**) curves for neat VAE, S1 (VAE/IFR), S2 (VAE/IFR/FA) and S3 (VAE/IFR/FA/KH403).

The cone calorimetry results of neat VAE and flame-retardant VAE samples are summarized in Table 3 and Figure 2. Compared with neat VAE (S0), all flame-retardant samples showed improved fire performance. As shown in Figure 2a, S0 exhibited a high peak heat release rate (pHRR_1_) of 660 kW/m^2^, indicating its rapid combustion. After the incorporation of IFR, the pHRR_1_ of S1 (VAE/IFR) decreased to 334 kW/m^2^, corresponding to a reduction of 49.39%. With the further addition of FA and FA/KH403, the pHRR_1_ values of S2 (VAE/IFR/FA) and S3 (VAE/IFR/FA/KH403) decreased to 303 and 246 kW/m^2^, representing reductions of 54.09% and 62.73%, respectively. These results indicate that IFR effectively suppressed the heat release of VAE, and the introduction of FA or FA/KH403 further enhanced this effect.

For the flame-retardant samples, a second heat release peak appeared during the middle-to-late combustion stage. The pHRR_2_ values of S1, S2, and S3 were 264, 190, and 217 kW/m^2^, respectively. Compared with S1, the pHRR_2_ values of S2 and S3 were reduced by 28.03% and 17.80%, suggesting that FA contributed to a more stable protective char layer and further delayed heat release. In terms of total heat release, the THR_250s_ of S0 was 58.6 MJ/m^2^, whereas those of S1, S2, and S3 decreased to 41.4, 38.6, and 40.0 MJ/m^2^, respectively. Among them, S2 showed the lowest THR value, indicating that the FA/IFR system was more effective in reducing the overall heat release. Notably, S3 exhibited the lowest pHRR_1_, suggesting better heat release suppression at the early combustion stage.

Smoke release is another important parameter for evaluating fire safety. As shown in Figure 2c and Table 2, S0 showed the highest peak smoke production rate (pSPR) of 0.145 m^2^/s. After adding IFR, the pSPR of S1 decreased to 0.079 m^2^/s. The pSPR values of S2 and S3 were 0.089 and 0.104 m^2^/s, respectively, slightly higher than that of S1 but still lower than that of S0. In terms of total smoke production (TSP), S2 exhibited the lowest value of 10.6 m^2^, which was reduced by 16.54% and 15.20% compared with S0 and S1, respectively. This result suggests that FA played a positive role in suppressing total smoke release. By contrast, S3 showed a higher TSP value of 14.0 m^2^, which may be related to incomplete combustion or changes in the char structure caused by the introduction of KH403. Although S1 had the lowest pSPR, S2 achieved a better balance between pSPR and TSP, indicating its more favorable overall smoke suppression performance.

The residual mass curves during cone calorimetry are shown in Figure S1. The residual weight after cone calorimetry further reflects the char-forming ability of the samples. Neat VAE left only 8.59% residue, confirming its poor char-forming capacity. The residual weight of S1 increased to 26.46% after the addition of IFR, while S2 reached the highest value of 30.90%. This result indicates that FA promoted char formation and improved the stability of the condensed phase. The residual weight of S3 was 22.87%, lower than that of S2 but still much higher than that of S0. The fire performance index (FPI = tign/pHRR_1_) was also calculated to evaluate fire hazard. The FPI values of S0, S1, S2, and S3 were 0.0288, 0.0539, 0.0561, and 0.0732 m^2^·s/kW, respectively. The highest FPI value of S3 indicates its lower fire hazard and stronger ability to suppress heat release at the initial combustion stage.

The CO/CO_2_ ratio was used to further evaluate the gas-phase combustion behavior. VAE/IFR25 showed the highest CO/CO_2_ ratio of 0.0973, mainly due to the sharp decrease in CO_2_ release from 1.619 to 0.637 and the increase in CO release from 0.043 to 0.062. This indicates that the IFR system suppressed the complete oxidation of volatile products, which is consistent with its flame-inhibition and condensed-phase barrier effects. After introducing FA, the CO/CO_2_ ratio decreased to 0.0819, and the CO release was reduced to 0.048, suggesting that FA improved the char barrier and partly alleviated incomplete combustion. For VAE/IFR20/FA3/KH2, the CO/CO_2_ ratio slightly increased to 0.0880, but its absolute CO release remained low at 0.043, while CO_2_ release decreased to 0.488. This suggests that more carbon was retained in the condensed phase rather than fully oxidized. Combined with the FRI results, VAE/IFR20/FA3/KH2 showed better early-stage heat shielding, whereas VAE/IFR20/FA5 was more favorable for char formation and total smoke suppression.

Overall, the cone calorimetry results confirm that the incorporation of flame retardants significantly improved the fire safety of VAE. The addition of KH403 further reduced the early-stage pHRR and gave the highest FPI value, but it also led to increased smoke production and a lower residual weight compared with S2. The underlying flame-retardant mechanism will be further discussed based on the structure and composition of the char residues.

### 3.3. TGA Analysis

**Figure 3:**
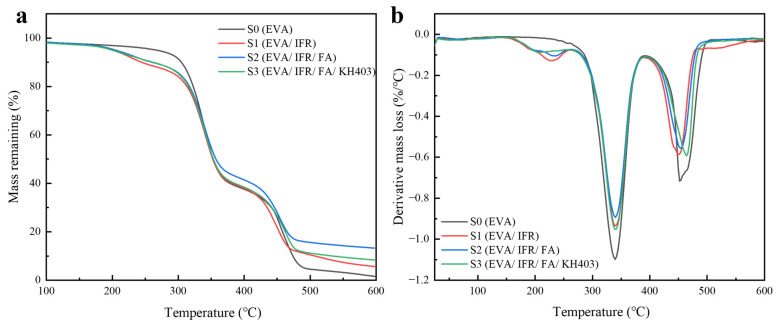
TGA (**a**) and DTG (**b**) curves of neat VAE and flame-retardant VAE samples (S1 (VAE/IFR), S2 (VAE/IFR/FA) and S3 (VAE/IFR/FA/KH403)).

The thermal degradation behavior of neat VAE and flame-retardant VAE samples was investigated under a nitrogen atmosphere, and the TG and DTG curves are shown in Figure 3. All samples exhibited similar degradation profiles, while the flame-retardant samples showed markedly increased char residues compared with neat VAE.

As shown in Figure 3a, neat VAE (S0) mainly underwent two degradation stages. The first stage occurred at approximately 240–360 °C, which was associated with the decomposition of vinyl acetate units and the release of volatile acetic acid. The second major degradation stage occurred in the range of 380–480 °C, corresponding to the thermal decomposition of polyethylene sequences in the polymer backbone. As listed in Table 4, the residual weight of S0 at 600 °C was only 1.54%, indicating the poor char-forming ability of neat VAE. After the incorporation of IFR, the char residue of S1 (VAE/IFR) increased to 5.62%, which was 264.94% higher than that of S0. This result suggests that the APP/MEL/PER-based IFR system promoted char formation during thermal degradation. With the further addition of FA, the residual weight of S2 (VAE/IFR/FA) increased significantly to 13.26%, representing a 135.94% increase compared with S1. This improvement indicates that FA played a positive role in promoting char formation and improving the thermal stability of the condensed phase. For S3 (VAE/IFR/FA/KH403), the residual weight was 8.31%, higher than that of S1 but lower than that of S2, suggesting that the introduction of KH403 did not further increase the final char yield under nitrogen. The TG curves obtained in air are shown in Figure S3. Neat VAE underwent rapid oxidative degradation above approximately 300 °C and was almost completely decomposed by 550 °C. In contrast, the flame-retardant samples showed slower mass loss and retained about 28–32% residue at 600 °C. Among them, S3 exhibited the highest residual mass, indicating that the IFR/FA/KH403 system improved char stability and reduced oxidative degradation under air atmosphere.

The DTG curves provide further information on the degradation behavior of the samples. Neat VAE showed two main mass loss peaks at 339 °C and 452 °C, corresponding to the decomposition of vinyl acetate units and the degradation of polyethylene segments, respectively. For the flame-retardant samples, an additional mass loss peak appeared in the lower temperature region at approximately 208–234 °C, which can be attributed to the early decomposition of the IFR components, especially the thermal decomposition of APP and the release of NH_3_ and H_2_O [36]. In this stage, phosphoric acid and polyphosphoric acid species generated from APP can promote dehydration and carbonization reactions. The lower Tmax1 of S3 may be related to the earlier decomposition or condensation reactions of the KH403-containing system. Meanwhile, the T_max3_ values of S2 and S3 were 455 °C and 464 °C, respectively, slightly higher than that of S0, indicating that FA and KH403 contributed to improving the thermal stability of the polymer matrix at higher temperatures.

The peak mass loss rate (PMLR) was also reduced after flame-retardant modification. The PMLR of S0 was −1.10%/°C, while those of S1, S2, and S3 decreased to −0.94, −0.89, and −0.95%/°C, corresponding to reductions of 14.55%, 19.09%, and 13.64%, respectively. Among all samples, S2 exhibited the lowest PMLR and the highest char residue, indicating the slowest thermal degradation rate and the strongest char-forming ability. These results are consistent with the cone calorimetry results, in which S2 exhibited lower heat release and higher residual weight.

### 3.4. Mechanical Properties

The mechanical properties of neat VAE and flame-retardant VAE samples are shown in Table 5. Neat VAE exhibited a tensile strength of 12.6 MPa, an elongation at break of 6.7%, and a plywood bonding strength of 0.88 MPa. After adding IFR and FA, these values decreased to 11.5 MPa, 4.3%, and 0.75 MPa for VAE/IFR/FA, respectively, indicating that the introduction of solid flame-retardant particles weakened the continuity of the VAE matrix and reduced the interfacial compatibility. In contrast, the addition of KH403 improved the tensile strength, elongation at break, and bonding strength to 12.4 MPa, 5.3%, and 0.84 MPa, respectively. This result suggests that KH403 enhanced the interaction between FA and the VAE/IFR matrix, thereby improving the mechanical integrity and practical usability of the flame-retardant adhesive.

### 3.5. Mechanism Analysis

Although VAE/IFR/FA performed better in char formation and total smoke suppression, VAE/IFR/FA/KH403 showed advantages in early-stage heat release suppression, FPI, and mechanical/bonding performance. Considering the practical use of VAE adhesives in coatings and plywood bonding, VAE/IFR/FA/KH403 was selected as a representative formulation for further mechanism analysis.

#### 3.5.1. Morphology of Char Residue

**Figure 4:**
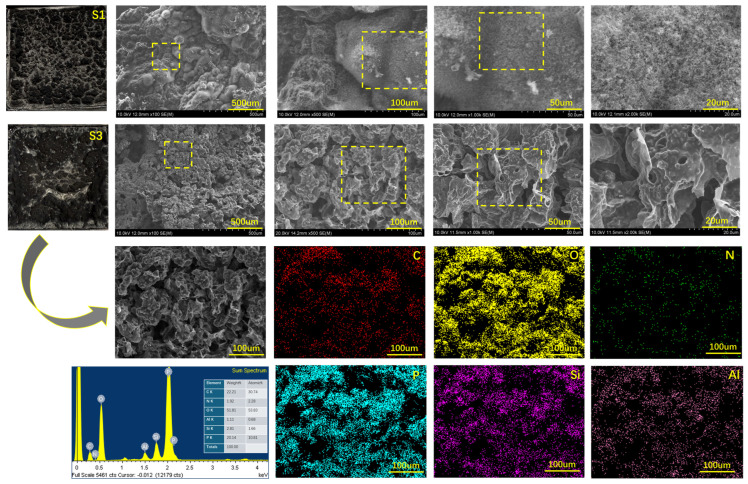
Digital images, SEM images and EDX spectra of char residues of S1 (VAE/IFR) and S3 (VAE/IFR/FA/KH403).

The digital photographs, SEM images of the char residues of S1 (VAE/IFR) and S3 (VAE/IFR/FA/KH403) after cone calorimetry, together with the EDS analysis of S3, are shown in Figure 4 The char residue of S1 (VAE/IFR) exhibited a swollen but relatively fragile structure, with visible cracks and discontinuous regions on the surface. This indicates that the APP/MEL/PER-based IFR system promoted char formation to some extent, but the resulting char layer was not sufficiently compact to act as an effective barrier during combustion.

The SEM images further confirmed the difference in char morphology between S1 and S3. For S1, the char surface was loose and broken, and obvious cracks and open pores could be observed at higher magnification. Such a porous and discontinuous structure would allow heat, oxygen, and volatile degradation products to pass through the char layer, thereby weakening its protective effect. In contrast, S3 (VAE/IFR/FA/KH403) formed a more continuous and compact char residue. The char surface showed a folded and interconnected structure, and the number of large cracks was clearly reduced. Although some pores were still present, the overall char layer was more integrated than that of S1. This improvement suggests that the introduction of FA and KH403 contributed to the formation of a stronger condensed-phase barrier.

EDS analysis was performed on the char residue of S3 to further examine its elemental composition. As shown in Figure 4, the char residue contained C, O, N, P, Si and Al, with contents of 22.21, 51.81, 1.92, 20.14, 2.81 and 1.11 wt%, respectively. The high contents of P and O indicate that phosphorus-containing species derived from IFR were retained in the condensed phase after combustion. These species could promote dehydration and carbonization reactions and contribute to the formation of a phosphorus-rich protective char. In addition, the presence of Si and Al confirmed that FA was incorporated into the residual char, while Si may also be associated with the contribution of KH403. These inorganic components can reinforce the char layer and improve its thermal stability and barrier performance.

The EDS mapping images of S3 showed that C, O, P, Si and Al were distributed throughout the char residue, suggesting that the phosphorus-containing char and inorganic components were integrated within the condensed phase. This organic–inorganic hybrid char structure is beneficial for improving the compactness and mechanical integrity of the protective layer. Combined with the SEM observations, these results indicate that FA and KH403 improved the quality of the IFR-derived char layer, leading to a more continuous and thermally stable barrier. This enhanced char structure can effectively reduce heat transfer, oxygen diffusion, and the release of combustible volatiles, thereby improving the flame-retardant performance of the VAE adhesive system.

#### 3.5.2. Raman Spectrum of Char Residue

**Figure 5:**
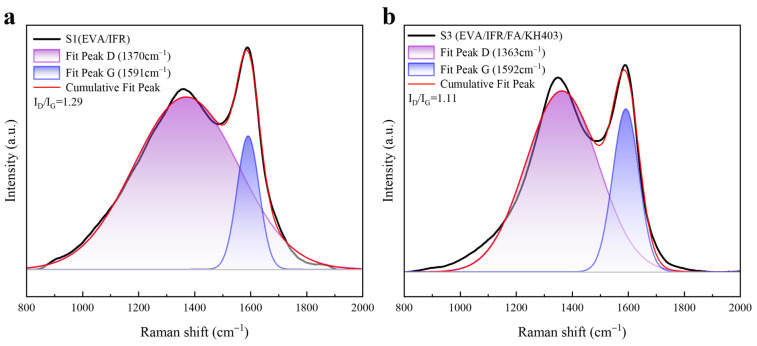
Raman spectra of S1 (VAE/IFR) (**a**) and S3 (VAE/IFR/FA/KH403) (**b**).

Raman spectroscopy was used to further evaluate the structural order of the char residues after combustion. As shown in Figure 5, both S1 (VAE/IFR) and S3 (VAE/IFR/FA/KH403) exhibited two characteristic bands at approximately 1350 and 1580 cm^−1^, corresponding to the D and G bands, respectively. The D band is associated with defect-induced vibrations of disordered carbon structures, while the G band is attributed to the in-plane stretching vibration of sp^2^-hybridized carbon atoms in graphitic structures. Therefore, the intensity ratio of the D band to the G band (ID/IG) is commonly used to assess the graphitization degree and defect density of carbonaceous residues.

The ID/IG value of S1 was 1.29, whereas that of S3 decreased to 1.11. The lower ID/IG value of S3 indicates a higher degree of graphitic ordering and fewer structural defects in the char residue. This result suggests that the incorporation of FA and KH403 promoted the formation of a more ordered and stable carbonaceous char during combustion. The improved char quality is beneficial for enhancing the barrier effect of the condensed phase, thereby suppressing heat transfer, oxygen diffusion, and the release of combustible volatiles. This finding is consistent with the SEM and XPS results, further confirming the positive role of FA/KH403 in strengthening the IFR-derived protective char layer.

#### 3.5.3. XPS Analysis of Char Residue

**Figure 6:**
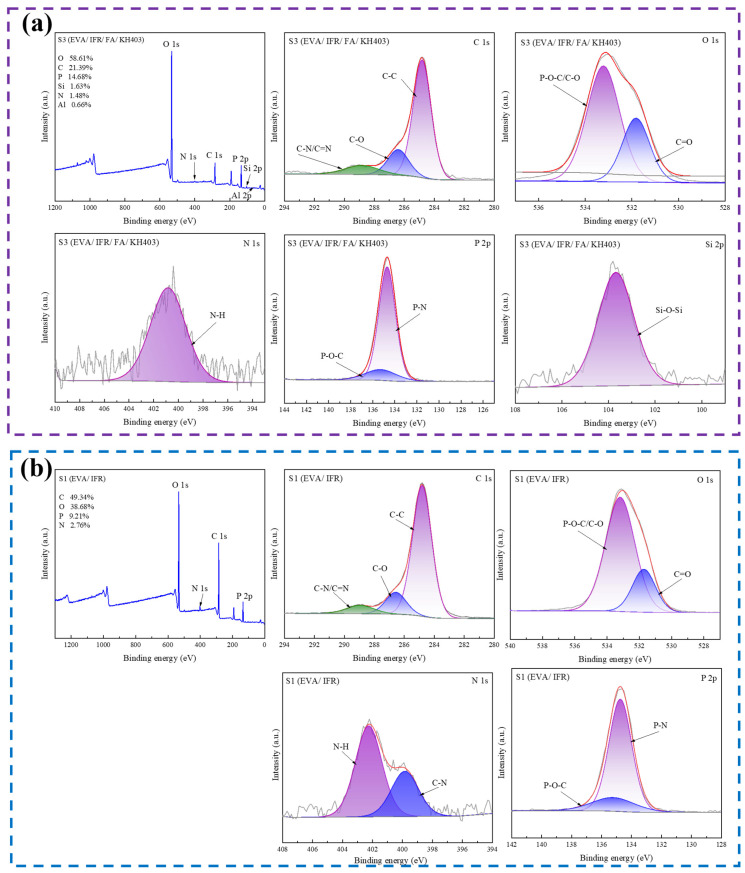
XPS survey spectra and high-resolution C 1s, O 1s, N 1s, P 2p, and Si 2p spectra of the char residues of S1 (VAE/IFR) (**b**) and S3 (VAE/IFR/FA/KH403) (**a**) after cone calorimetry.

X-ray photoelectron spectroscopy (XPS) was used to analyze the surface chemical composition of the char residues of S1 (VAE/IFR) and S3 (VAE/IFR/FA/KH403), as shown in Figure 6. The survey spectrum of S1 showed the presence of C, O, P and N, with relative contents of 49.34%, 38.68%, 9.21% and 2.76%, respectively. These elements mainly originated from the VAE matrix and the APP/MEL/PER-based IFR system. In comparison, the char residue of S3 contained O, C, P, Si, N and Al, with relative contents of 58.61%, 21.39%, 14.68%, 1.63%, 1.48% and 0.66%, respectively. The appearance of Si and Al in S3 confirmed the participation of FA and KH403 in the char residue. Meanwhile, the higher O and P contents suggested that more phosphorus- and oxygen-containing structures were retained on the char surface, which may contribute to the formation of a more thermally stable protective layer.

The high-resolution C 1s spectra of both S1 and S3 could be fitted into several components. The peak at around 284.6 eV was assigned to C-C/C-H bonds, corresponding to the carbon skeleton of the VAE matrix and carbonaceous char. The peak near 286.0 eV was attributed to C–O and/or C–N bonds, which may originate from the acetate groups in VAE, the decomposition products of IFR, and crosslinked structures formed during combustion. The component at approximately 288.5 eV was assigned to oxidized carbon species such as C=O/O-C=O. Compared with S1, the C content of S3 decreased markedly, while the relative contents of O and P increased, indicating that the char surface of S3 was enriched with inorganic and phosphorus-containing species rather than only carbonaceous residues.

In the O 1s spectra, the peak at approximately 531.8 eV was assigned to C=O and/or P=O groups, while the peak around 533.2 eV corresponded to C-O, P-O-C and P-O-P structures. These oxygen-containing groups indicate the formation of phosphate or polyphosphate structures in the condensed phase. For S3, the stronger oxygen signal was also related to the presence of Si-O bonds from FA and KH403. This result suggests that the FA/KH403-containing system favored the retention of inorganic oxygen-containing structures in the char residue, which could improve the barrier effect of the char layer.

The N 1s spectra reflected the contribution of nitrogen-containing components from the IFR system. For S1, the peaks located at around 400.0 and 402.0 eV could be attributed to C-N and N-H, respectively. These signals were associated with melamine and ammonium polyphosphate-derived structures. In S3, the N 1s signal was mainly concentrated near 400 eV, indicating that part of the nitrogen-containing species remained in the char residue after combustion. These nitrogen-containing structures may participate in the formation of a phosphorus-nitrogen-containing char network.

The P 2p spectra of S1 and S3 further confirmed the involvement of phosphorus-containing species in the condensed phase. The main peak around 134.0 eV can be assigned to phosphate-related structures, including P-O-P, P-O-C and/or P-N bonds, while the component near 135.0 eV is associated with more oxidized phosphorus species. These structures are generally derived from APP decomposition and subsequent dehydration and crosslinking reactions. The presence of P-O-C/P-O-P structures indicates that phosphorus-containing species promoted the formation of a crosslinked phosphate-rich char layer, which is beneficial for suppressing heat and mass transfer during combustion.

Notably, the Si 2p signal was only observed in S3. The peak at approximately 103.5 eV was attributed to Si-O-Si and/or Si-O-C bonds, corresponding to the silicate structure of FA and the interfacial structures formed by KH403. The presence of Si-containing species indicates that FA and KH403 were retained in the condensed phase and participated in the construction of the protective char layer. Such silicate-based structures can act as inorganic reinforcing components, improving the compactness and thermal stability of the char residue.

Overall, the XPS results show that the char residue of S1 mainly consisted of carbonaceous structures and phosphorus-nitrogen-containing species derived from IFR. After the introduction of FA and KH403, Si- and Al-containing inorganic components were incorporated into the char residue, and the surface became enriched with O- and P-containing structures. The coexistence of phosphate structures, nitrogen-containing species, and silicate networks indicates that FA/KH403 can cooperate with IFR to form a more stable organic-inorganic char structure. This result provides chemical evidence for the improved condensed-phase flame-retardant effect of the VAE/IFR/FA/KH403 system.

#### 3.5.4. Gas Phase Analysis

**Figure 7:**
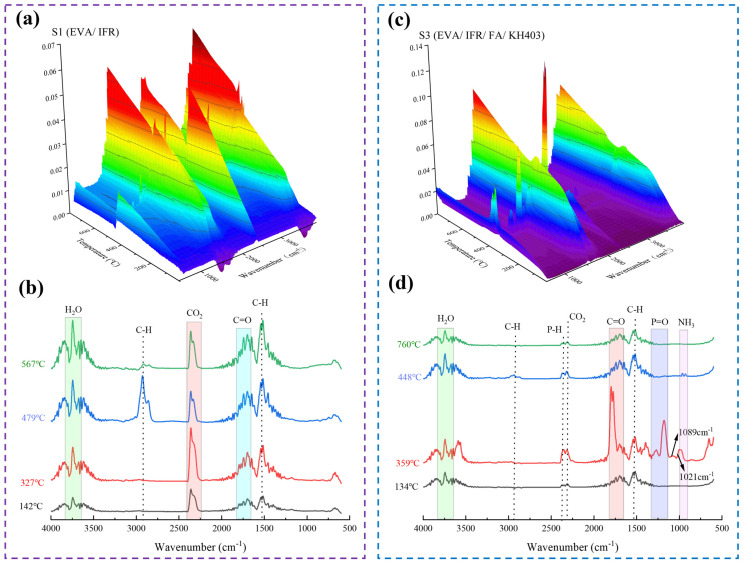
Characterization of pyrolysis gases of S1 (VAE/IFR) (**a**,**b**) and S3 (VAE/IFR/FA/KH403) (**c**,**d**).

TG-FTIR was used to analyze the volatile products released during the thermal degradation of S1 (VAE/IFR) and S3 (VAE/IFR/FA/KH403), so as to further clarify the gas-phase flame-retardant behavior. Figure 7 shows the three-dimensional FTIR spectra and the FTIR spectra collected at selected temperatures, which were chosen according to the main mass-loss stages in the DTG curves. For S1, several typical gaseous products were detected during pyrolysis. The absorption bands at approximately 3855 and 3752 cm^−1^ were assigned to H_2_O, while the bands at 2811–2970 cm^−1^ and around 1512 cm^−1^ were related to C-H-containing volatile fragments. The strong absorption near 2351 cm^−1^ was attributed to CO_2_, and the peaks at 1694 and 1735 cm^−1^ corresponded to carbonyl-containing compounds, such as acetic acid, aldehydes, ketones, or ester-derived degradation products. These signals indicate that the thermal degradation of VAE mainly involved deacetylation, chain scission, and the release of oxygen-containing volatile products. Compared with S1, S3 showed different gas evolution behavior. The absorption band around 1318 cm^−1^ can be assigned to P=O or P-O-containing species derived from the decomposition of the IFR system. These phosphorus-containing species may contribute to flame inhibition in the gas phase and also participate in the formation of phosphorus-rich char in the condensed phase. In addition, the bands at approximately 964 and 924 cm^−1^ were attributed to NH_3_ released from nitrogen-containing components such as APP and MEL. The release of NH_3_ can dilute combustible volatiles and oxygen in the flame zone, thereby contributing to the gas-phase flame-retardant effect.

It should be noted that Si-containing components in FA are mainly present as inorganic oxides or aluminosilicate structures. These species are difficult to volatilize during thermal degradation and therefore are not directly reflected in the TG-FTIR spectra. Their role is mainly associated with the condensed phase. As confirmed by the EDS and XPS results, Si and Al were retained in the char residue of S3, indicating that FA participated in the construction of the residual char layer. These inorganic components could reinforce the char structure, improve its thermal stability, and enhance its barrier effect. Moreover, the intensities of CO_2_ and carbonyl-containing volatile products released from S3 were lower than those of S1 at comparable degradation stages, suggesting that the introduction of FA and KH403 suppressed the release of volatile degradation products to some extent. This can be attributed to the improved condensed-phase barrier formed by the combined action of IFR, FA, and KH403, which limited the transfer of heat and volatile fragments. Therefore, the flame-retardant effect of S3 originated from both gas-phase dilution/flame inhibition by IFR-derived species and condensed-phase protection by phosphorus-rich char and Si/Al-containing inorganic structures.

Based on the cone calorimetry, TG-FTIR, SEM-EDS, XPS, and Raman results, the flame-retardant mechanism of the VAE/IFR/FA/KH403 system is proposed in Figure 7. During combustion, APP decomposes to generate phosphoric acid and polyphosphoric acid species, which catalyze dehydration and char formation, while MEL releases nonflammable NH_3_ to dilute combustible volatiles. FA introduces Si- and Al-containing inorganic components into the condensed phase, reinforcing the phosphorus-rich char layer and improving its thermal stability. KH403 may improve the interfacial interaction among FA, IFR, and the VAE matrix through Si-O-C/Si-O-Si structures, leading to a more continuous organic-inorganic protective layer. It should be noted that KH403 did not improve all fire-related parameters. Compared with S3, S2 showed higher char residue and lower total smoke production, indicating better char-forming and overall smoke-suppression effects. In contrast, S3 exhibited lower early-stage heat release, reduced initial smoke release, and the highest fire performance index. This suggests that KH403 mainly enhanced the early barrier effect of the char layer rather than simply increasing the final char yield. Therefore, the improved flame retardancy of the system results from the balance between char formation, char integrity, heat shielding, and smoke suppression.

## 4. Conclusions

In this study, fly ash (FA) was introduced into a conventional APP/MEL/PER intumescent flame-retardant system to prepare flame-retardant VAE emulsion adhesives. The results showed that FA reinforced the IFR-derived char layer and improved the char-forming and smoke-suppression performance of the VAE adhesive. Compared with neat VAE, the LOI value of the VAE/IFR/FA sample increased from 18.3% to 28.5%, and the UL-94 rating reached V-0. Cone calorimetry results also confirmed the improved fire safety, as the pHRR and THR were significantly reduced, and the residual char was increased. The role of FA was mainly reflected in promoting char formation and improving the stability of the condensed phase. The VAE/IFR/FA sample showed the highest char residue in both cone calorimetry and TGA tests, indicating that FA could cooperate with IFR to form a more effective protective char layer. SEM, EDS, XPS, and Raman analyses further demonstrated that the introduction of FA contributed to the formation of a compact organic–inorganic char structure containing phosphorus-rich carbonaceous species and Si/Al-containing inorganic components. This reinforced char layer could effectively inhibit heat transfer, oxygen diffusion, and the release of combustible volatiles. The addition of KH403 further adjusted the combustion behavior of the VAE/IFR/FA system. Although the VAE/IFR/FA/KH403 sample did not show the highest final char residue or the lowest total smoke production, it exhibited the lowest early-stage heat release and the highest fire performance index. This indicates that KH403 mainly improved the early barrier effect and heat-shielding ability of the char layer, rather than simply increasing the final char yield. Therefore, the flame-retardant performance of the system was governed by the balance among char formation, char integrity, heat release suppression, and smoke control. Overall, FA showed good potential as a low-cost synergistic additive for intumescent flame-retardant VAE emulsion adhesives. This work provides a feasible strategy for improving the fire safety of VAE adhesives while realizing the high-value utilization of industrial solid waste.

It should be noted that KH403 did not improve all fire-related parameters. Although VAE/IFR/FA showed higher char residue and lower TSP, VAE/IFR/FA/KH403 exhibited the lowest first pHRR and the highest FPI, indicating better early-stage heat shielding. Together with the improved tensile properties and plywood bonding strength, this suggests that KH403 mainly enhanced the interfacial compatibility and practical usability of the VAE/IFR/FA system rather than simply increasing the final char yield. Therefore, VAE/IFR/FA was more favorable for char formation and overall smoke suppression, whereas VAE/IFR/FA/KH403 provided a better balance between early fire protection and mechanical performance.

Future work should further optimize the dispersion of FA and its interfacial interaction with VAE/IFR systems, with the aim of reducing phosphorus loading while maintaining high flame-retardant efficiency. In addition, the bonding performance, water resistance, aging durability, and application performance in wood-based panels should be systematically evaluated to promote the practical use of this low-cost flame-retardant adhesive system.

## Figures and Tables

**Table 1:** Formula of neat VAE and flame retardant VAE samples.

Samples	VAE (wt.%)	IFR (wt.%)	FA (wt.%)	KH403 (wt.%)	P (wt.%)
Neat VAE (S0)	100	0	0	0	0
VAE/IFR (S1)	75	25	0	0	4.79
VAE/IFR/FA (S2)	75	20	5	0	3.84
VAE/IFR/FA/KH403 (S3)	75	20	3	2	3.84

**Table 2:** The LOI values and UL-94 ratings of neat VAE and flame-retardant VAE samples.

Samples	S0	S1	S2	S3
LOI/%	18.3 ± 0.1 d	±0.2 c	25.8 ± 0.1 b	26.3 ± 0.3 a
UL-94	NC	V1	V0	V0

Different letters followed the mean values means significant difference at 0.05 level (*p* < 0.05).

**Table 3:** Key data from the cone calorimeter test of neat VAE and flame-retardant VAE samples.

Samples	t_ign_ (s)	t_pHRR1_ (s)	pHRR_1_ (kW/m^2^)	t_pHRR2_ (s)	pHRR_2_ (kW/m^2^)	THR_250s_ (MJ/m^2^)	Residual Weight (%)	FPI (m^2^·s /kW)	FRI	pCO (%)	pCO_2_ (%)	pCO/pCO_2_ (%)	pSPR (m^2^/s)	TSP_200s_ (m^2^)
S0 (EVA)	19	106	660	/	/	58.6	8.59	0.0288	1	0.043	1.619	0.0266	0.145	12.7
S1 (EVA/IFR)	18	73	334	122	264	41.4	26.46	0.0539	2.65	0.062	0.637	0.0973	0.079	12.5
S2 (EVA/IFR/FA)	17	71	303	160	190	38.6	30.9	0.0561	2.96	0.048	0.586	0.0818	0.089	10.6
S3 (EVA/IFR/FA/KH403)	18	71	246	199	217	40.0	22.87	0.0732	3.72	0.043	0.488	0.0881	0.104	14.0

**Table 4:** Essential data of neat VAE and flame-retardant VAE samples in TGA under nitrogen atmosphere.

Samples	T_max_ 1 (°C)	T_−5 wt%_ (°C)	T_max_ 2 (°C)	T_max_ 3 (°C)	W_exp_ (%) 600 °C	PMLR (%/°C)
S0 (EVA)	/	312	339	452	1.54	−1.10
S1 (EVA/IFR)	229	317	339	452	5.62	−0.94
S2 (EVA/IFR/FA)	234	320	340	455	13.26	−0.89
S3 (EVA/IFR/FA/KH403)	208	319	339	464	8.31	−0.95

**Table 5:** Mechanical data of neat VAE and flame-retardant VAE samples.

Samples	Tensile Strength (MPa)	Elongation at Break (%)	Bonding Strength in Plywood (MPa)
S0 (EVA)	12.6 ± 0.3	6.7 ± 0.3	0.88 ± 0.03
S1 (EVA/IFR)	11.9 ± 0.4	4.8 ± 0.2	0.76 ± 0.02
S2 (EVA/IFR/FA)	11.5 ± 0.2	4.3 ± 0.2	0.75 ± 0.02
S3 (EVA/IFR/FA/KH403)	12.4 ± 0.3	5.3 ± 0.3	0.84 ± 0.03

## Data Availability

The data presented in this study are available on request from the corresponding author.

## Supplementary Materials

The following supporting information can be downloaded at: https://www.mdpi.com/article/10.3390/polym18151884/s1, Figure S1: Mass loss of sample during combustion process under cone calorimeter test, Figure S2: SEM and EDX pictures of FA, Figure S3: TGA curves of neat VAE and flame-retardant VAE samples under air.
