# Supplementary material for: Flame Retardancy of Vinyl Acetate–Ethylene Emulsion Adhesives Modified with Intumescent Flame Retardants and Fly Ash
Source: Polymers (Basel). 2026 Jul 31;18(15):1884. doi: 10.3390/polym18151884 (PMC13468526; doi:10.3390/polym18151884)
Supplement: Supplementary file 1 [file polymers-18-01884-s001.zip › polymers-4411119-supplementary.pdf]

Article

Flame Retardancy of Vinyl Acetate–Ethylene Emulsion Adhesives Modified with Intumescent Flame Retardants and Fly Ash

Qianqian Wang [1,2], Jing Luo [3], Yan Sun [2,*], Wei Liu [4], Xucai Wang [2] and Peng Jiang [5,*]

1 National Key Laboratory of Strength and Structural Integrity, School of Aeronautic Science and Engineering, Beihang University, Beijing 100191, China; wqq2024@126.com
2 Shandong Institute of Nonmetallic Materials, Jinan 250031, China; wangxucai01@163.com
3 China Nuclear Power Engineering Co., Ltd., Beijing 100840, China; luojing@cnpe.cc
4 Sichuan Fire Research Institute of Ministry of Emergency Management, Chengdu 610036, China; weiliu20141202@126.com
5 Research Institute of Wood Industry, Chinese Academy of Forestry, Beijing 100091, China
* Correspondence: wosyh@163.com (Y.S.); jiangpeng@caf.ac.cn (P.J.)

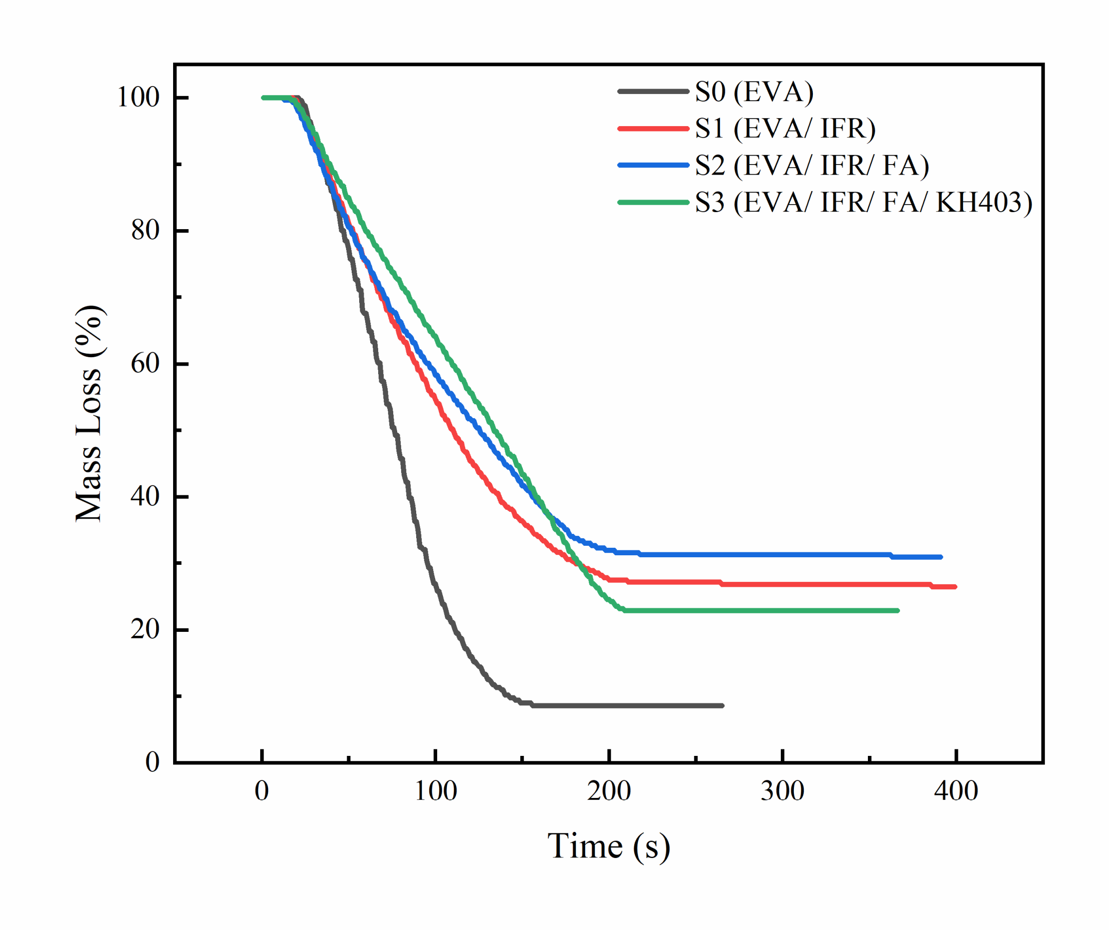

Figure S1 Mass loss of sample during combustion process under cone calorimeter test.

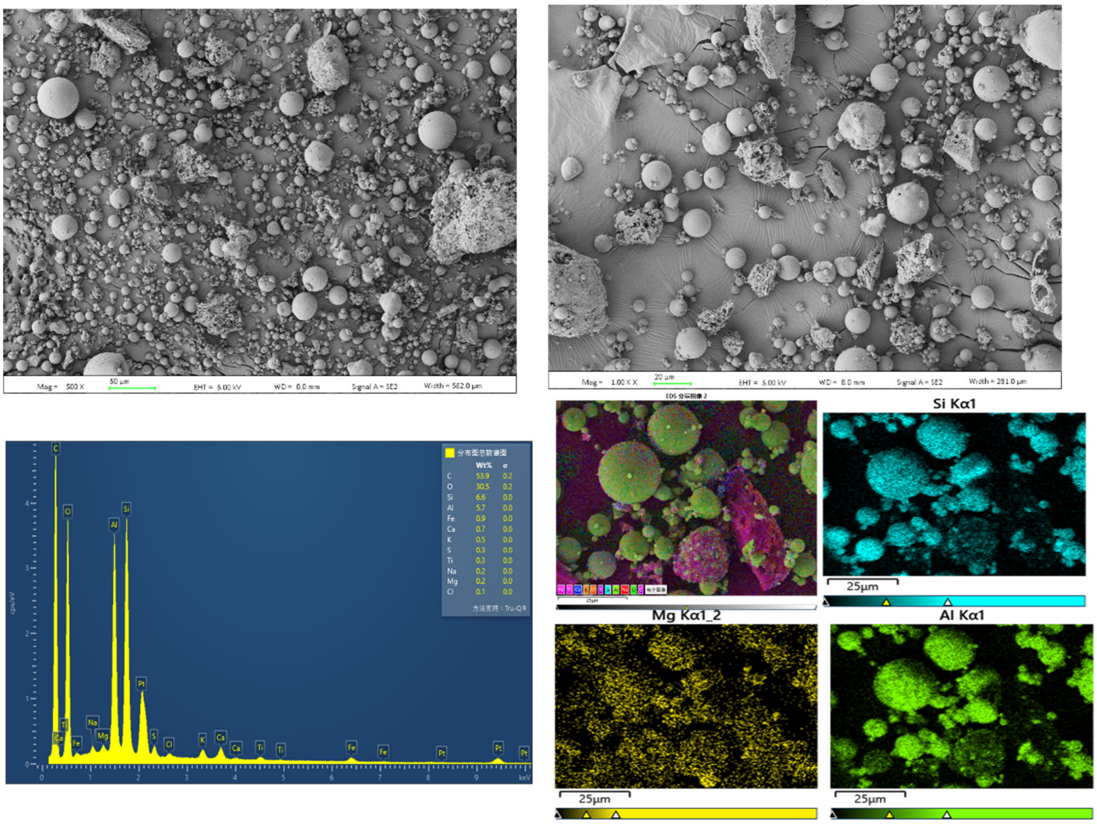

Figure S2 SEM and EDX pictures of FA.

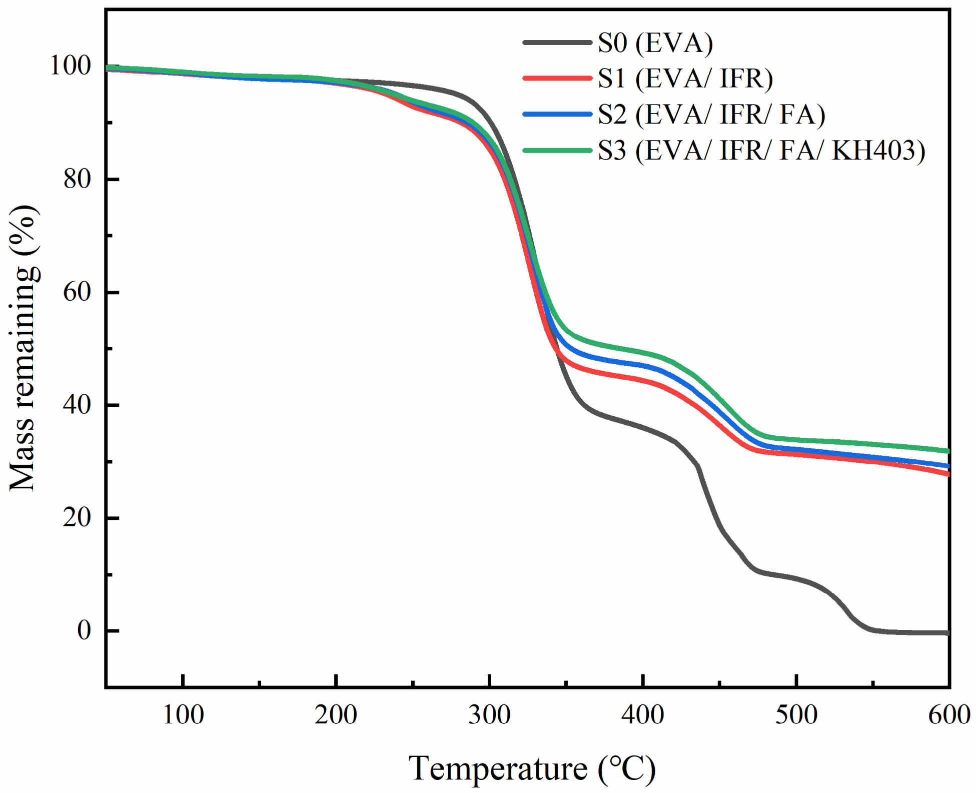

Figure S3 TGA curves of neat VAE and flame-retardant VAE samples under air.
